# Surgical Treatment of Primary Intracardiac Myxoma: 20-Year Experience in “Shahid Modarres Hospital”—A Tertiary University Hospital—Tehran, Iran

**DOI:** 10.1155/2015/303629

**Published:** 2015-01-26

**Authors:** Zahra Ansari Aval, Hamid Ghaderi, Hassan Tatari, Mahnoosh Foroughi, Seyedeh Adeleh Mirjafari, Mohammad Forozeshfard, Kamal Fani, Isa Khaheshi

**Affiliations:** ^1^Lung Transplantation Research Center, National Research Institute of Tuberculosis and Lung Diseases (NRITLD), Shahid Beheshti University of Medical Sciences, Tehran, Iran; ^2^Cardiovascular Research Center, Department of Cardiovascular Surgery, Shahid Modarres Hospital, Shahid Beheshti University of Medical Sciences, Tehran, Iran; ^3^Brain and Spinal Injury Research Center (BASIR), Tehran University of Medical Sciences, Tehran, Iran; ^4^Department of Anesthesiology, Semnan University of Medical Sciences, Semnan, Iran; ^5^Department of Anesthesiology, Shahid Modarres Hospital, Shahid Beheshti University of Medical Sciences, Tehran, Iran; ^6^Cardiovascular Research Center, Shahid Modarres Hospital, Shahid Beheshti University of Medical Sciences, Tehran, Iran

## Abstract

Although cardiac tumors are not common they may vary in terms of race and surgical approach in different countries. *Method*. Patients data of 20 years was collected and evaluated in the “Shahid Modarres Hospital”—a tertiary university hospital—Tehran, Iran. *Results*. 42 patients with cardiac myxoma (all cases in 20 years) were included in study, 17 males and 25 females, age difference: 13 to 76 years (mean 50.6). Most of patients were in functional classes I, II. 35 patients complained of dyspnea and 3 patients had embolic events. 97.6% of tumors were primary (41 patients) and one tumor was recurrent (2.4%), 85.7% of tumors (36 cases) were located in LA, and 88.1% of tumors (37 cases) were pediculated. 40 patients (95%) had one tumor. In 22 patients (52.3%) after tumor resection septal defects were repaired primarily while in 18 patients (42.8%) the defects were repaired with pericardial patch and In one patient, tumor resected without any septal defect. Mean tumor size was about 5.22 cm (range of 2.2 to 8.2 cm). Postoperatively, 33 patients discharged from hospital without any complication. *Discussion*. The research reveals that patients' age and gender were similar to that of other studies in other countries while tumor's incidence seems to be higher. 3 patients were diagnosed after remote embolic event and one patient was diagnosed after MI reflecting relatively high tumor complications and late diagnosis. *Conclusion*. In our study mean time from diagnosis to operation was too long. The patients had more preoperative embolic events and complication. However, size of myxoma and location of that was as same as its rate in the other literature. As recommendation we suggested that in all patients with vague chest pain or remote embolic events cardiac myxomas should be ruled out.

## 1. Introduction

Primary cardiac tumors are rare ones. Their annual incidence is 5 per million among people of 30 to 60 years old [1]. They comprise 0.3% of all cardiac operations [2]. Although their prevalence is low, they can lead to severe permanent disabilities. They are cause of death if they are diagnosed late or situated in critical locations.

Crafoord reported first cardiac myxoma diagnosis and successful operation by CPB in 1955 [3]. Tumors clinical manifestation and symptom's severity depend on tumor size and its location in heart.

## 2. Method

This study is descriptive and has been conducted by collecting data of patients admitted in Tehran's Shahid Modarres Hospital—a tertiary university hospital—with diagnosis of cardiac myxoma from 1994 to 2014. Patients' data then got categorized according to their age, gender, clinical manifestations, diagnostic modalities, preoperative functional class, preoperative and postoperative arrhythmia, serologic abnormalities, waiting time to operation, hospital stay, operative findings, concurrent operations, complications, and patients' outcome.

## 3. Results

44 patients with cardiac myxoma underwent operation in 20 years (total cardiac tumor candidates for this study were 52 from which 44 patients had cardiac myxoma and other 8 had other cardiac tumors). Two patients were excluded from study due to lack of data and finally 42 cases were included in the study. 17 (40.4%) and 25 (59.6%) were male and female cases, respectively, and patients' mean age was 50.6 ± 17 (rang of 13 to 76) (Table 1).

Mean time from diagnosis to operation was 5.8 days (range from 1 to 48 days) and mean postoperative hospital stay was 12.7 days (range 5 to 32 days). Patients' most common complaints were dyspnea in 23 patients (54.8%), dyspnea with palpitation in 12 cases (28.5%), palpitation in 5 patients (11.9%), weight loss in 9 patients (21.4%), and 3 patients admitted in hospital with embolic events (Table 2).

Six patients were in NYHA class I (14.3%), 17 patients in class II (40.5%), 14 patients in class III (33.3%), and 4 patients in NYHA class IV (9.5%). Three patients had a history of cerebral emboli (CVA or TIA) (Table 3).

Transthoracic echocardiography (TTE) was the primary diagnostic modality in all patients; indeed in 22 patients (52.4%) TTE was the only diagnostic tool but in 5 patients (11.9%) transoesophageal echocardiography (TEE) was used as well. In 19 patients (45.23%) coronary angiography was used to rule out concurrent coronary artery disease (Table 1). In addition to echocardiography and angiography, CT-scan was used in one patient.

Laboratory findings were charted as mean WBC: 8294 ± 280 (normal range: 4300–12900), mean HCT: 38.56 ± 5.38% (normal range; 26%–47%); ESR and CRP were also checked; however, in some patients these data had not been checked, so to analyze these data was out of value.

Almost all tumors were primary (41 patients, 97.6%) compared to one recurrent case (2.4%) (Table 3).

Tumors were primary in 41 patients (97.6%) and recurrent in one patient (2.4%) (Table 3). Tumors' location was in left atrium (LA) in 36 cases (85.6%), both atria in 2 cases (4.8%), and right atrium (RA) in 3 patients (7.2%) and right ventricle (RV) in one patient (2.4%). Tumors were pediculated in 37 patients (90%) and sessile in 3 patients (7.2%). Tumors were single in 40 patients (95.2%) and double in one case.

After tumor removal, septal defect was repaired primarily in 22 cases (52.4%) while for 18 cases (42.8%) pericardial patch was applied. In one patient, tumor was resected without any residual defect in septum. Also, for one case tumor had been located in right ventricular outflow tract with extension into pulmonary valve leaflets for which in addition to the tumor right ventricular septal partially resected and pulmonary valve was repaired.

Along with tumor removal mitral valve replacement was performed in two patients (in one case due to rheumatic mitral stenosis and in one case due to severe mitral regurgitation). Mitral annuloplasty was done in two patients as well (due to severe MR) and one patient underwent CABG concurrent with tumor resection.

Patients were followed up for a mean time of 48.8 months (range 1–82 months).

33 patients (78.4%) were discharged postoperatively from hospital without any complication. Postoperative complications were

1. there was post-CPR loss of vision in ICU in one patient (after cardiac arrest);
2. there was heart block in two cases, a complete and a partial one which were managed with and without pacemaker, respectively;
3. because of huge tumor size and chronic illness one patient experienced congestive heart failure;
4. secondary to tumor emboli two patients developed cerebral complications (hemiplegia and aphasia);
5. in one patient that postembolic coma was developed having many late complications including low LOC, prolonged mechanical ventilation, tracheotomy, blue toe, and bed sore;
6. one month after discharging from hospital one patient died due to noncardiac problems (DIC, hepatorenal syndrome and loss of consciousness);
7. Preoperative CVA and pneumonia resulted in postoperative death in one case (Table 4).

Discharging from hospital 33 patients (78.4%) had NSR (normal sinus rhythm). On the other hand, some others suffered from different dysrhythmia. AF: 4 patients, partial heart block: 3 patients, PAT with heart block: 1 patient and one patient had complete heart block.

Mean tumors' size was 5.22 ± 1.6 cm (range of 2.2 to 8.2 cm).

## 4. Discussion

Cardiac myxoma is the most important cardiac tumor. Its annual incidence is 5 in one million people. Each cardiac surgery center encounters one to two of this rare disease in year [3].

There were 42 cardiac myxomas in our hospital in last 20 years (incidence rate: 2.1 per year). Meanwhile in Middle East no precise statistic records about this tumor prevalence are at hand. However, there is a report of 35 cases in 5-year period (7 cases in each year) in Tabriz [4], which may be attributed to single referral cardiac surgery center in that city.

Zheng et al. reported 66 cardiac myxoma cases within 26 years in China (2.5 cases per year) [5]. One study reported 41 cases in 19 years in Greece (0.48 cases per year) [6]. Another report from Australia stated 24 cases in 30 years (0.8 cases per year) [7]. So far, the largest reported series is attributed to Xue et al. from China, posing 211 myxoma tumors in 18 years (11.72 cases per year) [8].

All referral literatures emphasize female predominance [3, 8] and in our study there was 1.47 female/male ratio. Also, this ratio was reported as 1.33 in Shahid Madani Hospital in Tabriz [9]; in contrast with this recent trend in Greece and China [6, 8] female predominance has been changed to male predominance (male/female rate in Greece: 26/15 and in China 48/18).

These recent trends show a change in gender predominance which necessitates further study. According to many literatures disease clinical manifestations are evident in 3rd to 6th decades of life [3, 10]. In our study presentations were mostly reported in the same period (mean patients' age: 50.62 years). In Tabriz it was 52 years [9] and it has been mentioned to be 45 to 54 years in many other countries [5, 6, 10]. The youngest case in our study was 13 and oldest one was 76. Kirklin believed myxoma has not been seen and reported in infancy but Pasaoglu reported one myxoma case in a 35-day-old infant in Turkey [11]. Also, there are reports of a 3-year-old child with cardiac myxoma by Zheng et al. [5] and another 4-year-old child by Rebollar et al. [12].

In our study mean time from diagnosis to operation was 5.83 days (range of 1 to 48 days) and most operations were performed in one or two days after diagnosis.

Since the disease is naturally lethal and it requires urgent cardiac operation, there was no report of waiting time to operation in most cardiac surgical centers (8–10% of patients die while waiting for operation) [3, 5].

Common causes of delay in operation are patients' fear of operation, waiting for more diagnostic modalities such as coronary angiography, CT scan and TEE, and finally long waiting list in public hospitals [13]. In our study, mean hospital stay was 12.7 days (range of 5 to 32 days) which is comparable to reported results of one study with mean ICU stay of 2.3 ± 0.8 days and mean hospital stay of 7.9 ± 1.8 days [5].

Most common clinical symptoms in this study were dyspnea, dyspnea with palpitation and chest pain which were similar to other studies [3–6]. Although rate of embolic events has been reported to be about 5% in most literatures, in our study this rate was higher (7.1%). In Tabriz study, embolic events rate was 8.6% and 2.4% in Samanidis study [6, 9] for which we cannot explain the reason of differences in embolic events rate. It is not clear whether all patients with low level of consciousness are evaluated for cardiac tumors or all embolectomy specimens are sent for pathologic evaluation.

In all patients primary diagnostic modality was TTE. For 5 of them additionally TEE was conducted as well. Depending on patient's age, coronary angiography was applied to evaluate atherosclerosis (in 19 patients) leading to one concurrent CABG and myxoma operation. Although TEE can diagnose small tumors of 1–3 mm diameter, waiting for TEE can delay operation up to two weeks. So, it is not clear whether delay for TEE is beneficial for patient or not; also it is not certain if this delay favors or changes operation method.

Except one recurrent case (2.4%) who had a familial cancer history all myxomas were primary. Nearly 5% of myxomas are familial (autosomal dominant), they are mainly younger patients, and there is not gender predominance in this group [3, 10].

In 20% of them (familial group), tumor originates from atrium and ventricle. This group have abnormal DNA chromosomal genotype pattern [3, 6, 8]. In most literatures, tumors were found in LA (75%) and in RA (10–20%) and in ventricles in remaining cases (3) [3–5, 8, 11]. In our study tumors were found in LA (85.6%) and in both atria (4.8%) and in RA (2.4%). Mean tumor diameter was 5.22 cm which is similar to that of other literatures (mean diameter of 5-6 cm) [3, 10]. In the study of China mean tumor diameter was about 0.3–2.5 cm and in the study of Greece it was about 5.1 cm [5, 6].

There is no general agreement about repairing septal defect after tumor resection. Many surgeons repair it with artificial patches such as Dacron [5, 12, 14, 15] and others repair it with pericardial patch.

We prefer to repair septal defect with pericardial patch. In our center, septal defects were repaired with pericardial patch in 42.8% and with primary approach in 52.4% of cases. In one case with myxoma located on ventricular septum tumor was excised with partial septal resection [16].

In some occasions other cardiac operations are concurrently essential. We experienced a concurrent CABG, one pulmonary valve repair, two mitral valve repairs, and two mitral valve replacements. There are reports of concurrent CABG [17] and CABG with MVR in addition to myxoma removal [5].

Like other literatures, we witnessed postoperative arrhythmia after septal removal (21.42%) [3, 5, 13].

While average hospital mortality is around 5% [3, 5, 10] we observed that as 2.4%, one case with preoperative critical general condition (the patient was admitted in hospital with CVA and then developed pneumonia). Recent studies have reported near zero mortality [5, 6] which may be contributed to access to equipped referral hospital.

## 5. Conclusion

Myxoma tumors' prevalence in Iran was the same as that in other countries in the Middle East; however, its rate is less than that in East of Asia and more than that in Europe. These recent results indicate a change gender predominance, mean time from diagnosis to operation was too long. The patients had more preoperative embolic events and preoperative complications. In terms of size and location it was approximately parallel to other literatures.

AS vague chest pain and remote embolic events can raise doubt about myxoma existence. We recommended that in all patients with these findings myxoma should be ruled out.

## Figures and Tables

**Table 1:** Preoperative patients' information from 1994 to 2014 diagnosed with myxoma in Shahid Modarres Hospital.

Presentation (1994–2014)	Parameter	%
Gender	Male	17 (40.4%)
	Female	25 (59.6%)

Mean age		50.6 ± 17
	Range	13–76

Age	10–19 years	1 (2.4 %)
	20–29 years	3 (7.1%)
	30–39 years	5 (11.9%)
	40–49 years	6 (14.2%)
	50–59 years	15 (35.7%)
	60–69 years	9 (21.6%)
	70–80 years	3 (7.1%)

Diagnostic method	TTE + TEE + Angio	4 (9.5%)
	TTE + Angio	14 (33.3%)
	TTE + TEE	1 (2.4%)
	TTE	22 (52.4%)
	TTE + Angio + CT	1 (2.4%)

Functional class	Functional class I	6 (14.2%)
	Functional class II	17 (40.6%)
	Functional class III	14 (33.3%)
	Functional class IV	4 (9.5%)
	Unknown	1 (2.4%)

TTE: transthoracic echocardiography; TEE: transoesophageal echocardiography; Angio: angiography; CT: computed tomography.

**Table 2:** Clinical presentation (signs and symptoms) of patents diagnosed with myxoma from 1994 to 2014 in Shahid Modarres Hospital.

Clinical presentation (1994–2014)	%
Chest pain	3 (7.1%)
Dyspnea	23 (54.8%)
Dyspnea with fever and weight loss	1 (2.4%)
Dyspnea with palpitation	12 (28.5%)
CVA with hemiplegia	1 (2.4%)
TIA with IHD	1 (2.4%)
TIA with hemiparesia	1 (2.4%)
Fatigue	10 (23.8%)
Acute myocardial ischemia	1 (2.4%)
Palpitation	5 (11.9%)
Syncope	1 (2.4%)
Weight loss	9 (21.4%)
Peripheral edema	2 (4.8%)
Arthralgia	1 (2.4%)
Incidentally	1 (2.4%)
Unknown	1 (2.4%)

TIA: transient ischemic attack; CVA: cerebrovascular accident; IHD: ischemic heart disease.

**Table 3:** Specification of operation procedures performed for patents diagnosed with myxoma from 1994 to 2014 in Shahid Modarres Hospital.

Operating information	1994–2014	
Location	Left atrium	36 (85.6%)
	Right atrium	3 (7.2%)
	Left and right atrium	2 (4.8%)
	Right ventricle	1 (2.4%)

Kind of tumor	Primary	41 (97.6%)
	Recurrent	1 (2.4%)

Pediculate or sessile	Pediculate	37 (90%)
	Sessile	3 (7.2%)
	Unknown	2 (4.8%)

Operating technique	Primary repair	22 (52.4%)
	Repair with pericardial patch	18 (42.8%)
	Myomectomy	1 (2.4%)
	Unknown	1 (2.4%)

Combination operation	CABG	1 (2.4%)
	Repair of pulmonary valve	1 (2.4%)
	Mitral valve replacement	2 (4.8%)
	Mitral annuloplasty	2 (4.8%)

Tumor size	5.22 ± 1.6 cm, range: 2.2 to 8.2 cm	

CPB time	85.4 ± 31 min	

XC time	55.6 ± 30 min	

CABG: coronary artery bypass graft, CPB: cardiopulmonary bypass, XC: aortic cross clamp.

**Table 4:** Patients' well-being in discharge time diagnosed with myxoma from 1994 to 2014 in Shahid Modarres Hospital.

Secle	1994–2014
No secle	33 (78.4%)
Blue toe + CVA + tracheostomy	1 (2.4%)
Right hemiparesia	1 (2.4%)
Right hemiparesia with aphasia	1 (2.4%)
Visual loss	1 (2.4%)
Heart failure	1 (2.4%)
Complete heart block	1 (2.4%)
PAT with heart block	1 (2.4%)
Discharge and death after 1 month	1 (2.4%)
Hospital mortality	1 (2.4%)

Total	42
